# Fecal microbiota transplantation in ulcerative colitis: mucosal immune mechanisms and precision microbiota therapy

**DOI:** 10.3389/fimmu.2026.1888117

**Published:** 2026-07-20

**Authors:** Enhui Lu, Miao Zhang, Yuanyang Zhao, Hai Meng, Zhixuan Xu

**Affiliations:** 1 Department of Gastroenterology, Binhai County People’s Hospital, Yancheng, China; 2 Department of General Surgery, Binhai County People’s Hospital, Yancheng, China

**Keywords:** Donor–recipient matching, fecal microbiota transplantation, gut microbiota, microbiota–host interaction, mucosal immunity, precision microbiota therapy, Th17/Treg balance, ulcerative colitis

## Abstract

Fecal microbiota transplantation (FMT) has emerged as an investigational microbiota-targeted strategy for ulcerative colitis (UC), aiming to restore dysbiotic gut ecosystems and microbiota–host homeostasis. Mechanistic studies suggest that FMT may reshape microbial community structure, enrich short-chain fatty acid-producing bacteria, remodel bile acid and tryptophan–aryl hydrocarbon signaling, enhance epithelial barrier integrity, and regulate mucosal immunity, including Th17/Treg balance, IgA-associated responses, macrophage reprogramming, IL-10/IL-22 signaling, and neutrophil extracellular trap formation. Randomized controlled trials indicate that FMT can induce clinical and endoscopic remission in selected patients with UC, particularly when administered through the lower gastrointestinal route and using multi-donor or optimized regimens. Current guidelines do not recommend conventional FMT as routine therapy for UC outside clinical trials, reflecting the heterogeneity and low certainty of available evidence. However, its efficacy appears limited in moderate-to-severe or biologic-refractory disease, underscoring the importance of host inflammatory burden and recipient ecological receptivity. Personalized strategies, including donor–recipient functional matching, dietary modulation, combination therapy, and next-generation microbiota therapeutics, may improve response and safety but require prospective validation. This review integrates microbiota–metabolite–barrier–immune mechanisms with clinical evidence to define where FMT may be useful, where its benefit appears limited, and how future studies can move the field toward precision microbiota therapy in UC.

## 1 Introduction

Ulcerative colitis (UC) is a chronic, relapsing inflammatory disorder of the colonic mucosa. Recent global epidemiological data indicate that inflammatory bowel disease, including UC, is evolving across distinct regional stages, with prevalence accumulating in early industrialized regions and incidence rising in many newly industrialized regions (1). Although its incidence has stabilized or declined in many Western countries since 1990, it has continued to rise in newly industrialized regions of Asia, Africa, and South America, where rapid socioeconomic transition and westernized lifestyles have reshaped disease epidemiology (2). A forward-looking analysis by Kaplan (3) projected that early industrialized countries, already in the compounding prevalence stage, may see inflammatory bowel disease (IBD) prevalence exceed 1% of the population within the next decade and approach prevalence equilibrium by the 2040s. Although biologic agents, including anti-TNF antibodies, vedolizumab, and ustekinumab, together with small-molecule drugs such as tofacitinib and upadacitinib, have substantially expanded therapeutic options, many patients still experience primary non-response, secondary loss of response, or failure to achieve sustained one-year clinical remission (4). This persistent therapeutic gap has intensified interest in the gut microbiota as a central and potentially modifiable node in UC pathogenesis.

Gut microbial dysbiosis is increasingly recognized as an important contributor to UC pathogenesis. Patients with UC commonly exhibit reduced microbial diversity, depletion of short-chain fatty acid (SCFA)-producing bacteria, including *Faecalibacterium prausnitzii* and *Roseburia hominis*, and enrichment of potentially pathogenic taxa such as adherent-invasive *Escherichia coli* (5, 6). This dysbiotic state may promote chronic inflammation by impairing epithelial barrier integrity and activating aberrant mucosal immune responses, processes that intersect with host genetic susceptibility (7). Restoring microecological homeostasis through microbiota-targeted interventions has therefore emerged as a biologically plausible therapeutic strategy. However, microbiota restoration should not be equated with proven clinical efficacy. FMT trials in UC have produced heterogeneous results, and FMT should be viewed as an investigational microbiota-directed strategy rather than an established standard therapy for UC.

Fecal microbiota transplantation (FMT) involves the transfer of intestinal microbiota from a healthy donor into the gastrointestinal tract of a recipient to re-establish a functionally resilient microbial ecosystem. In recurrent *Clostridioides difficile* infection, fecal microbiota–based therapies are effective in preventing recurrence and have been incorporated into clinical practice guidelines (8). In UC, multiple meta-analyses over the past decade have suggested that FMT can induce clinical and endoscopic remission in selected patients, particularly those with mild-to-moderate disease (9, 10). However, efficacy remains heterogeneous, with outcomes varying according to donor characteristics, preparation method, administration route, treatment intensity, and patient population (11). These observations underscore the need to move beyond an empirical “one-size-fits-all” approach toward a more personalized and mechanism-driven paradigm.

Recent reviews, including that by Nagayama et al. (12), have highlighted the promise of precision microbiota therapy in IBD, particularly donor–recipient matching and individualized treatment strategies. Important gaps nevertheless remain. Existing reviews have not fully integrated emerging mechanistic domains, such as ferroptosis and extracellular vesicle-mediated effects, with the broader microbiota–metabolite–immune network. Nor have they sufficiently examined donor–recipient matching as an independent translational strategy, the manufacturing and quality-control barriers that limit clinical implementation, or the implications of recent boundary-defining trials published in 2025–2026.

This review addresses three interconnected themes. First, we synthesize current evidence on the molecular and cellular mechanisms of FMT in UC, including microbiota reconstitution, metabolic reprogramming, epithelial barrier repair, and immune regulation, and integrate these pathways within a systems-level framework that emphasizes the challenge of causal inference. Second, we critically appraise the clinical evidence, with particular attention to recent trials that help define the therapeutic boundaries of FMT rather than simply confirm its efficacy. Third, we examine translational strategies, including donor–recipient functional matching, manufacturing standardization, combination therapy, predictive biomarkers, and next-generation microbiota therapeutics. By distinguishing established evidence from unresolved uncertainty, this review aims to provide a balanced framework for understanding the current role and future development of FMT in UC. Unlike previous reviews that primarily summarized the efficacy or biological mechanisms of FMT in IBD, this review focuses on how recent mechanistic and clinical evidence can be used to define the therapeutic boundaries of FMT in UC and guide its transition from empirical transplantation toward precision microbiota therapy.

### 1.1 Search strategy and study selection

This narrative review was based on a literature search of PubMed from inception to April 2026 using combinations of the following terms: “fecal microbiota transplantation,” “FMT,” “ulcerative colitis,” “inflammatory bowel disease,” “mechanism,” “mucosal immunity,” “donor matching,” “personalized microbiota therapy,” “next-generation microbiota therapeutics,” and “clinical trial.” The initial search identified more than 500 records. After title, abstract, and full-text assessment, more than 100 articles were considered directly relevant, and approximately 90 key studies were finally included. Reference lists of relevant articles and recent reviews were also manually screened. Priority was given to randomized controlled trials, systematic reviews and meta-analyses, human mechanistic studies, major consensus statements or guidelines, and well-controlled preclinical studies with clear translational relevance. Recent landmark studies, including RESTORE-UC and STOP-Colitis, were prioritized because they directly informed therapeutic boundaries, delivery-route selection, donor strategy, or trial design. Because this article is a narrative review rather than a systematic review, no formal PRISMA-based screening, risk-of-bias assessment, evidence grading, or meta-analysis was performed.

## 2 Mechanisms of action

Although clinical studies suggest that FMT may benefit selected patients with UC, the biological mechanisms underlying this effect remain incompletely defined. Several key questions remain unresolved: does FMT act mainly by reshaping microbial community structure, restoring microbial metabolic functions, or both? Is sustained engraftment of live donor bacteria required for therapeutic efficacy? And are the molecular and immunological changes observed after FMT true causal mediators, or merely correlates of inflammation resolution?

**Figure 1:**
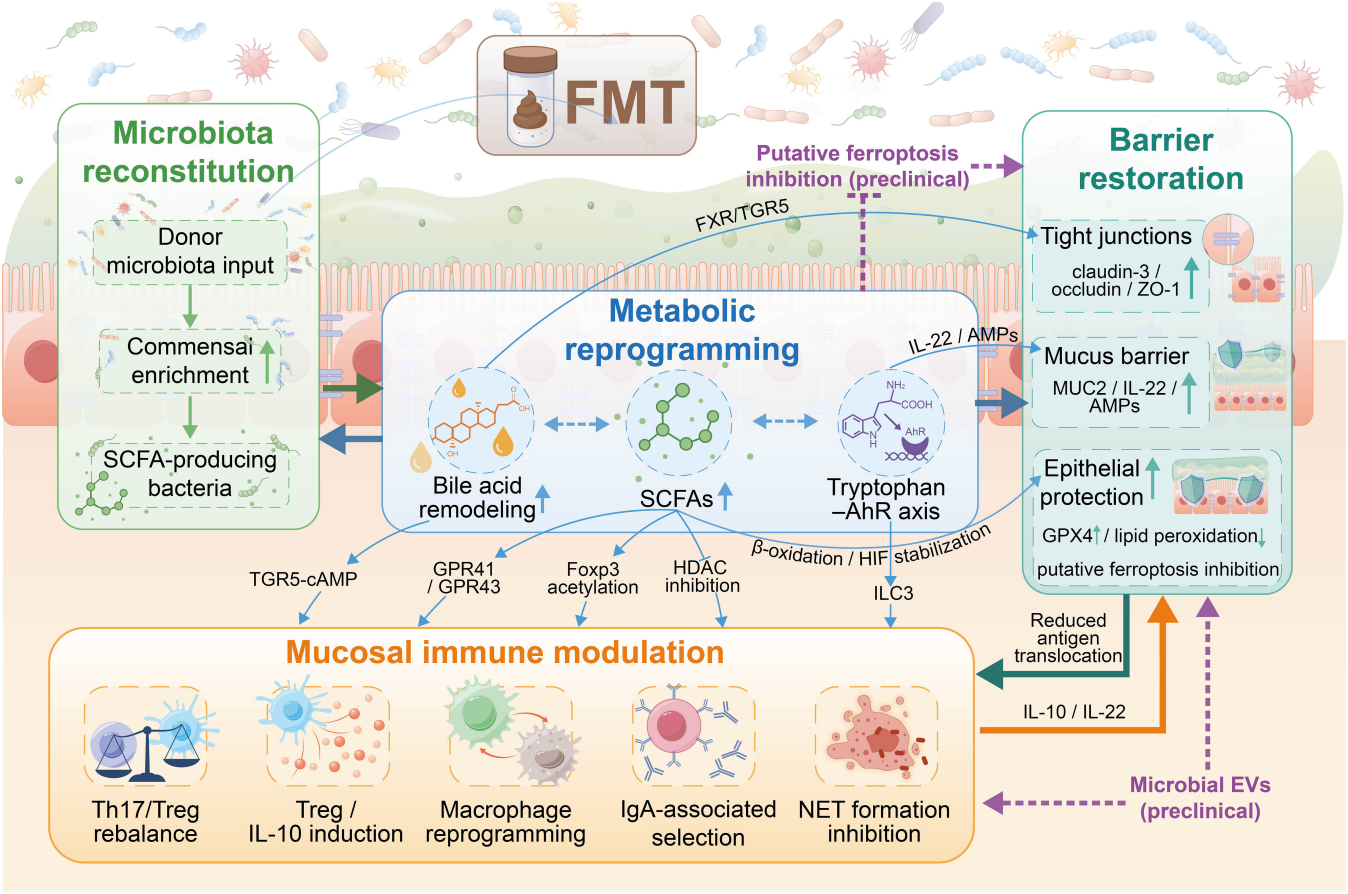
Proposed mechanisms of fecal microbiota transplantation in ulcerative colitis. FMT may promote microbiota reconstitution by introducing donor microbiota, enriching commensal bacteria, and increasing SCFA-producing bacteria. These ecological changes may drive metabolic reprogramming involving bile acid remodeling, SCFA production, and tryptophan–AhR signaling. The resulting microbial and metabolic signals may contribute to epithelial barrier restoration through regulation of tight junctions, mucus barrier function, and epithelial protection, while also modulating mucosal immune responses, including Th17/Treg rebalance, Treg/IL-10 induction, macrophage reprogramming, IgA-associated microbial selection, and inhibition of NET formation. Improved barrier integrity may reduce antigen translocation, whereas immune mediators such as IL-10 and IL-22 may further support barrier repair. Putative ferroptosis inhibition and microbial extracellular vesicle-mediated effects are shown as emerging, largely preclinical mechanisms. This image was originally created by the authors and contains no previously published material.

FMT is generally thought to act through an interconnected microbiota–metabolite–immune network, but the causal relationships within this network remain difficult to establish. This section reviews four major mechanistic dimensions: microbiota reconstitution, metabolic reprogramming, epithelial barrier repair, and immune regulation. Particular emphasis is placed on findings supported by human evidence or robust experimental validation. Because much of the mechanistic literature still derives from animal models, *in vitro* systems, or compound-plus-FMT experimental designs, its generalizability to human UC should be interpreted with caution. Compared with SCFA metabolism, bile acid remodeling, barrier repair, and immune regulation, evidence linking FMT to ferroptosis inhibition and extracellular vesicle-mediated effects remains preliminary and largely preclinical. The proposed mechanistic framework is summarized in **Figure 1**.

### 2.1 Microbiota reconstitution and metabolic reprogramming

The most immediate consequence of FMT is modulation of the recipient gut microbiota. Emerging evidence suggests that therapeutic success may depend not only on donor strain engraftment, but also on recipient-side ecological factors.

Pinto et al. (13) reported in a longitudinal study of 22 patients with UC receiving four rounds of FMT that patients achieving combined clinical and endoscopic remission retained a higher proportion of their original resident microbial species, whereas non-responders, despite initial donor-strain colonization, rapidly lost these strains. This finding challenges the simple “super-donor” model and suggests that recipient microbial resilience may be an important determinant of post-FMT outcomes. However, given the small sample size and limited sequencing resolution, this observation should be interpreted as exploratory rather than definitive.

Consistent with this concept, Zhao et al. (14) reported in a retrospective cohort of 96 patients with IBD that persistence of 38 recipient-native bacterial genera was associated with FMT responsiveness. Using these taxa as predictive features, a random forest model identified a high-probability subgroup in a small prospective validation cohort, in which remission at week 14 was numerically higher than in the low-probability subgroup. Although promising, this model remains vulnerable to overfitting and has not yet been validated in an independent external cohort.

Together, these studies suggest that the recipient intestinal ecosystem may critically shape FMT efficacy. Rather than acting simply through wholesale replacement of the recipient microbiota, FMT may function by perturbing a dysbiotic ecosystem toward a more stable and inflammation-resistant state. This conceptual shift has therapeutic implications: strategies that enhance ecological receptivity, such as dietary modulation, mucosal protection, or targeted preconditioning, may improve FMT response, but remain largely untested. Although donor-dependent responses have been observed in some studies (15), these recipient-centered findings argue against a universal “super-donor” model and suggest that donor efficacy should be interpreted in relation to recipient ecological receptivity rather than as an intrinsic donor property alone.

At the compositional level, enrichment of SCFA-producing bacteria is among the most consistently reported post-FMT alterations. A systematic review screening 3,755 publications found that butyrate-producing taxa from the *Lachnospiraceae* and *Oscillospiraceae* families were characteristic of responders (16). However, most available evidence is associative and cannot determine whether enrichment of butyrate producers drives therapeutic benefit or merely reflects inflammation resolution. This causal uncertainty is a recurrent limitation of the mechanistic literature and is discussed further in Section 3.1.

Among butyrate-producing bacteria, *Faecalibacterium prausnitzii* is of particular interest. Qin et al. (17) showed that *F. prausnitzii* may enhance intestinal IgA responses through production of inecalcitol, with its abundance inversely correlated with disease severity. Nevertheless, whether IgA enhancement directly mediates clinical improvement after FMT remains unproven. In an oral FMT randomized controlled trial, Raich et al. (18) used deep metagenomic sequencing and identified *Clostridium, Alistipes*, and the L-citrulline biosynthesis pathway as response-associated features. These findings suggest that FMT efficacy may involve transfer or restoration of specific strain-level metabolic functions rather than wholesale microbial replacement. However, as with many metagenomic associations, the L-citrulline pathway may represent a marker of a healthier ecosystem rather than a true effector mechanism. Functional validation through targeted metabolite supplementation, pathway inhibition, or strain-level mechanistic studies remains lacking.

#### 2.1.1 Metabolic hubs: SCFAs, bile acids, and tryptophan–AhR

Metabolic reprogramming can be regarded as a functional readout of microbiota reconstitution. Xu et al. (19) reported that fecal butyrate concentrations increased after FMT and found *in vitro* that butyrate enhanced microbial α-diversity under anaerobic culture conditions. These findings raise the possibility of a self-reinforcing loop in which FMT enriches butyrate-producing bacteria, increases butyrate availability, and thereby supports the expansion of additional beneficial microbes. However, whether such a feedback loop operates *in vivo* remains uncertain. Clarifying this issue will require longitudinal metabolomic sampling with greater temporal resolution than is currently available in most FMT trials.

Bile acid metabolism has received increasing attention as another key metabolic pathway influenced by the gut microbiota. Patients with UC typically exhibit an excess of primary bile acids and a reduction in secondary bile acids, a pattern linked to depletion of Clostridium cluster XIVa bacteria with 7α-dehydroxylase activity. FMT has been associated with partial restoration of secondary bile acid profiles, including deoxycholic acid and lithocholic acid, which may suppress NF-κB signaling and strengthen epithelial barrier function through FXR and TGR5 activation (20, 21). However, FXR and TGR5 differ in their intestinal and hepatic expression patterns, and systemic FXR activation may carry metabolic risks, including dyslipidemia. Moreover, the long-term metabolic consequences of FMT-induced bile acid remodeling have not been systematically evaluated. Given the potential genotoxicity of sustained high concentrations of secondary bile acids, their therapeutic window remains undefined (22).

The tryptophan–aryl hydrocarbon receptor (AhR) axis provides another important link between microbial metabolism, epithelial protection, and immune regulation. Clinical observations suggest that high baseline *Candida* abundance may be associated with FMT response, with post-FMT reduction in *Candida* accompanied by increased AhR ligand levels (23). One plausible explanation is that fungi influence bacterial tryptophan metabolism through substrate competition or inhibitory metabolites, thereby altering AhR activity. However, this association is based on limited clinical data, and whether the Candida–AhR relationship is causal remains unclear. Experimental studies further support the relevance of microbial tryptophan metabolism to AhR/IL-22 signaling, including models in which microbiota-targeted interventions increased indole derivatives and activated AhR-dependent protection (24, 25). The strongest causal evidence comes from a compound-plus-FMT murine model in which AhR blockade abolished the protective effect, supporting AhR as a downstream mediator in that specific context (25). Whether AhR activation is similarly required for conventional FMT efficacy in human UC remains unresolved. Taken together, these metabolic changes should be interpreted primarily as response-associated signatures rather than validated causal mediators in human UC. Their direction, magnitude, and durability may vary across sampling matrices, analytical platforms, sampling time points, diet, disease activity, and treatment regimens.

### 2.2 Intestinal barrier repair

The intestinal barrier comprises epithelial tight junctions, the mucus layer, and regulated epithelial cell death pathways that together maintain mucosal integrity. FMT has been proposed to support barrier repair through effects on these interconnected components. The following sections summarize the available evidence, while emphasizing the major limitations that constrain mechanistic interpretation, particularly the limited functional validation in human UC.

#### 2.2.1 Tight junctions

Luu et al. (26) performed transcriptomic profiling of colonic mucosal biopsies from UC patients treated with FMT and observed that 75 genes were significantly altered in the FMT group (q < 0.05), compared with only three genes in the placebo arm. Enriched pathways included tight junctions, calcium signaling, and xenobiotic metabolism. These findings provide human transcriptomic evidence that FMT may influence tight-junction-related pathways in the colonic mucosa and that these molecular changes are associated with clinical remission.

Animal studies provide additional protein-level support. In DSS-induced colitis models, microbiota transferred from donors exposed to specific dietary or natural-product interventions increased colonic expression of claudin-3, occludin, and ZO-1 and attenuated inflammatory injury (27, 28). These findings suggest that a reshaped microbial community can enhance tight junction protein expression in experimental colitis. However, because FMT was embedded within compound-intervention protocols rather than tested as an isolated intervention, the independent contribution of FMT itself cannot be clearly separated.

A key limitation is that human evidence remains largely confined to transcriptomic associations. Protein-level validation and direct functional measures of barrier integrity are still lacking in most FMT trials. Common functional assessments, such as transepithelial electrical resistance (TEER) or *in vivo* permeability tests including the lactulose/mannitol ratio, are rarely incorporated into clinical studies (29). Therefore, whether FMT-induced molecular changes translate into genuine functional barrier restoration remains uncertain. Future trials incorporating direct measurements of barrier integrity as secondary endpoints would substantially strengthen the mechanistic evidence base.

#### 2.2.2 Mucus layer and goblet cells

The mucus barrier depends largely on the synthesis and secretion of MUC2 mucin by goblet cells. In animal models, microbiota transferred from donors treated with specific prebiotic or polysaccharide interventions has been shown to promote goblet cell recovery, upregulate MUC2 expression, restore Th17/Treg balance, and attenuate colitis severity (30, 31). These studies provide proof-of-concept that intervention-shaped microbiota can transfer mucus barrier protection through FMT-like approaches in experimental colitis.

However, the translational relevance of these findings remains limited. Most available evidence comes from animal models using a “compound or natural product → microbiota alteration → FMT transfer” design, in which FMT is not tested as an independent intervention. No clinical study has directly evaluated the effects of FMT on goblet cell populations or MUC2 expression in UC patients. Whether FMT promotes goblet cell recovery directly through microbial metabolites or indirectly by reducing epithelial stress and inflammation remains unresolved. Addressing this question will require goblet-cell-specific and mucus-layer-related readouts in human studies.

#### 2.2.3 Ferroptosis

Ferroptosis, a regulated form of cell death driven by iron-dependent lipid peroxidation, has been implicated in intestinal epithelial injury in IBD. Several experimental studies have indirectly linked microbiota modulation or FMT-like transfer to ferroptosis inhibition. Peng et al. (32) isolated the polysaccharide TA2–1 from *Tremella aurantialba* and found that it upregulated colonic claudin-1 and ZO-1, suppressed lipid peroxidation, and modulated GPX4 in DSS-induced colitic mice; FMT experiments suggested that these protective effects were transmissible through the microbiota. Cheng et al. (33) similarly reported that phlorizin inhibited ferroptosis in a microbiota-dependent manner. These findings suggest that FMT may suppress intestinal epithelial ferroptosis indirectly through microbiota-mediated metabolic remodeling in specific compound-plus-FMT experimental settings.

Importantly, not all anti-ferroptotic effects are microbiota-dependent. Wei et al. (34) showed that astaxanthin alleviated colitis by inhibiting ferroptosis through the Nrf2/GPX4 axis, whereas FMT from astaxanthin-treated donors failed to reproduce the protective effect. This discordance highlights the need to distinguish microbiota-dependent from microbiota-independent mechanisms when discussing ferroptosis in the context of FMT.

Direct evidence in human UC remains lacking. To date, post-FMT changes in core ferroptosis markers, such as GPX4, ACSL4, and lipid peroxidation products, have not been systematically examined in intestinal mucosa from UC patients. Given the growing interest in ferroptosis as a therapeutic target in IBD, this represents an important gap in the translational evidence base. Therefore, ferroptosis should be regarded as an emerging preclinical hypothesis in the context of FMT rather than an established mechanism in human UC.

#### 2.2.4 Cross-cutting limitations

The barrier studies reviewed above share several recurrent limitations. First, much of the mechanistic evidence comes from animal experiments in which FMT is embedded within “compound + FMT” protocols and is not tested as an isolated intervention (27, 28, 30–33). Second, human validation remains largely restricted to transcriptomic associations, whereas protein-level confirmation and direct functional measures of barrier integrity, such as TEER or *in vivo* permeability testing, are rarely incorporated into FMT trials (29). Third, post-FMT changes in ferroptosis markers in human UC remain unexamined (32–34).

A related unresolved issue is the temporal relationship between structural and functional barrier recovery. Tight junction or MUC2-related molecular changes may occur earlier than complete functional barrier restoration, which likely requires epithelial regeneration and sustained resolution of mucosal inflammation. Without longitudinal sampling that integrates biopsy-based protein expression, permeability testing, and endoscopic healing, current studies cannot determine whether FMT-induced molecular changes translate into durable functional barrier repair. Future mechanism-driven trials should therefore incorporate direct barrier readouts and repeated sampling to move from molecular association toward functional causation.

### 2.3 Mucosal immune regulation

To better distinguish mucosal immune mechanisms from downstream correlates of inflammation resolution, this section separates adaptive T-cell responses, IL-22/ILC3-mediated epithelial immune repair, IgA-associated microbial selection, macrophage reprogramming, and NET formation. For each mechanism, we indicate whether the supporting evidence derives mainly from human studies, animal models, gnotobiotic experiments, or *in vitro* systems.

#### 2.3.1 Adaptive T-cell responses and the Th17/Treg axis

Rebalancing of adaptive T-cell responses is one of the most frequently proposed immunological mechanisms of FMT in experimental colitis. In DSS-induced and microbiota-dependent animal models, FMT or transfer of intervention-shaped microbiota has been associated with reduced Th1/Th17 responses, expansion of Foxp3^+^ regulatory T-cell populations, increased IL-10 production, and restoration of the Th17/Treg balance (31, 35, 36). These findings support a biologically plausible model in which microbial community remodeling may shift the mucosal immune environment away from effector inflammation and toward regulatory repair.

The strongest causal evidence for this axis remains preclinical. Lima et al. (37) identified an IgA-coated *Odoribacter splanchnicus* in UC patients who responded to FMT. In subsequent gnotobiotic experiments, this strain induced Foxp3^+^RORγt^+^ Treg cells and IL-10 production in germ-free mice. This study provides an important example of a responder-associated bacterium with experimentally validated immunomodulatory capacity.

However, the human evidence remains associative. The enrichment of responder-associated strains in patients does not prove that FMT-induced Th17/Treg rebalancing mediates clinical remission. Nor has it been shown that T-cell changes precede endoscopic or clinical improvement in human UC. Therefore, the Th17/Treg axis should be interpreted as a biologically plausible and experimentally supported mechanism, rather than as a proven mediator of FMT efficacy in patients. Future trials should incorporate paired mucosal immune profiling, such as flow cytometry, single-cell transcriptomics, or spatial immune mapping, to determine whether T-cell rebalancing is a driver of response or a downstream consequence of inflammation resolution.

#### 2.3.2 IL-22/ILC3-mediated epithelial immune repair

The IL-22/ILC3 axis provides a mechanistic link between microbial metabolism, epithelial repair, and mucosal immunity. IL-22 acts mainly on intestinal epithelial cells rather than immune cells. It can promote antimicrobial peptide production, epithelial regeneration, mucus-associated defense, and barrier restoration. Group 3 innate lymphoid cells (ILC3s) are an important mucosal source of IL-22 and may therefore connect microbial signals with epithelial immune repair. Recent evidence further supports the AhR/IL-22 axis as a microbiota-sensitive pathway through which tryptophan-derived ligands can regulate IL-22 production in CD4+ T cells and ILC3s, thereby contributing to epithelial repair and mucosal barrier protection (38).

In the context of FMT, this axis is most closely linked to microbial tryptophan metabolism and AhR signaling. Clinical observations suggest that FMT response may be associated with changes in fungal and bacterial tryptophan metabolism, including increased AhR ligand availability after reduction of Candida abundance (23). Experimental studies further support the relevance of microbiota-derived indole metabolites, AhR activation, and IL-22-associated epithelial protection in colitis models (24, 25). In one compound-plus-FMT murine model, AhR blockade abolished the protective effect, supporting AhR as a downstream mediator in that specific experimental setting (25).

However, direct evidence that conventional FMT activates an IL-22/ILC3 repair program in human UC remains limited. Most support comes from animal models, compound-plus-FMT designs, or mechanistic inference from microbial metabolite studies. Human FMT trials rarely quantify mucosal ILC3 populations, epithelial IL-22 responses, antimicrobial peptides, or spatial relationships between IL-22-producing cells and repairing epithelium. Thus, the IL-22/ILC3 axis should be regarded as a biologically plausible candidate mechanism linking microbial metabolic reprogramming to epithelial immune repair, but not yet as a validated mediator of FMT efficacy in human UC.

#### 2.3.3 IgA-associated microbial selection

IgA-associated microbial selection provides a mucosal immune perspective on FMT response. Secretory IgA is not only involved in pathogen neutralization. It can also regulate microbial localization, limit epithelial contact, and shape the persistence of selected commensal bacteria within the intestinal niche. In UC, IgA-coated bacterial profiling may therefore help identify microbes that are actively engaged by the mucosal immune system rather than microbes that are merely present in stool. In this review, “IgA-associated microbial selection” is used as a conceptual term referring to IgA-linked mucosal selection or persistence of microbes, whereas “IgA-coated” refers specifically to bacteria identified by IgA-coating-based approaches, such as IgA-seq or related profiling methods.

The most relevant evidence comes from Lima et al. (37), who identified an IgA-coated *Odoribacter splanchnicus* in UC patients who responded to FMT. This human finding is associative, but it is mechanistically informative because the same strain was subsequently tested in gnotobiotic mice. In that model, *O. splanchnicus* induced Foxp3^+^RORγt^+^ Treg cells and IL-10 production, supporting a direct immunoregulatory capacity of this responder-associated bacterium.

These findings suggest that IgA-based profiling may help connect clinical response, microbial strain identity, and mucosal immune function. However, IgA coating should not be interpreted as proof of benefit. It may reflect protective immune containment, but it may also mark bacteria exposed to an inflamed mucosal environment. Moreover, the causal immunological effect of *O. splanchnicus* was demonstrated in gnotobiotic mice, not directly in FMT-treated patients. Future studies should integrate IgA-seq, strain-level metagenomics, mucosal immune phenotyping, and clinical outcomes to determine whether IgA-associated bacteria are predictors, mediators, or markers of FMT efficacy.

#### 2.3.4 Macrophage reprogramming

Macrophage reprogramming may represent an innate immune mechanism through which microbiota remodeling attenuates mucosal inflammation. Here, reprogramming refers to a shift from pro-inflammatory M1-like programs toward less inflammatory, regulatory, or tissue-repair-associated macrophage states. This term is preferable to a simple “macrophage polarization” framework because intestinal macrophage states are dynamic and are shaped by microbial products, local metabolites, epithelial injury, and tissue-repair cues. Recent studies further emphasize that inflammatory-to-reparative macrophage transitions are closely linked to immunometabolic remodeling, including changes in oxidative phosphorylation, AMPK-related signaling, lactate metabolism, and glutamine metabolism (39).

In experimental colitis, microbiota-dependent signaling has been associated with reduced macrophage infiltration, decreased expression of pro-inflammatory mediators such as TNF-α, IL-1β, and iNOS, and partial restoration of regulatory or repair-associated immune programs (40). Microbial metabolites, including SCFAs and secondary bile acids, may contribute to macrophage reprogramming by modulating NF-κB signaling, inflammasome activation, and epithelial–myeloid crosstalk. More broadly, innate immune sensing pathways such as cGAS-STING may also shape macrophage activation states (41), but whether this pathway is modified by FMT in human UC remains unknown.

However, direct evidence that FMT induces macrophage reprogramming in human UC remains limited. Most clinical FMT studies have not included paired mucosal myeloid-cell profiling, macrophage subset mapping, single-cell analysis, or functional assays of macrophage cytokine production before and after treatment. Therefore, macrophage reprogramming should be considered a plausible innate immune pathway that requires validation in human mucosal tissue, rather than a proven mediator of clinical remission after FMT.

#### 2.3.5 Neutrophils and NET formation

Neutrophil extracellular trap (NET) formation is an emerging innate immune process relevant to mucosal injury in UC. NETs consist of extracellular DNA, histones, myeloperoxidase, neutrophil elastase, and other granular proteins released by activated neutrophils. Although NETs contribute to antimicrobial defense, excessive or persistent NET formation may amplify epithelial injury, expose immunostimulatory nuclear material, and sustain mucosal inflammation. Recent UC-focused evidence suggests that excessive NET formation may contribute to inflammatory amplification and mucosal barrier disruption (42).

Microbiota-dependent regulation of NET formation has begun to attract attention in experimental colitis. In particular, secondary bile acids modified by Odoribacter splanchnicus have been reported to alleviate colitis by suppressing NET formation (43). This finding provides a plausible link between microbiota-derived metabolites and neutrophil-driven mucosal inflammation. However, this evidence remains preclinical and should not be interpreted as proof that conventional FMT suppresses NET formation in human UC.

Future FMT studies should determine whether NET-associated changes occur before clinical response or merely reflect reduced inflammatory burden after treatment. Useful readouts may include citrullinated histone H3, myeloperoxidase–DNA complexes, neutrophil elastase, and tissue-based NET imaging. Until such data are available, NET suppression should be regarded as a promising but unvalidated candidate mechanism of FMT-associated mucosal immune regulation.

#### 2.3.6 Evidence hierarchy and unresolved causality

Taken together, the mucosal immune effects attributed to FMT in UC vary substantially in evidentiary strength. Human evidence is strongest at the associative level, including responder-associated microbial features, IgA-associated taxa identified by IgA-coating-based profiling, metabolomic changes, mucosal transcriptomic signals, and clinical correlations. Animal and gnotobiotic models provide stronger mechanistic support for selected pathways, including Treg induction, IL-10 production, AhR/IL-22-associated epithelial protection, macrophage inflammatory attenuation, and suppression of NET formation. *In vitro* studies further support receptor-level and cell-specific mechanisms, but they cannot establish whether these pathways mediate clinical remission after FMT.

This hierarchy has important implications for interpretation. Immune changes observed after successful FMT may either contribute to remission or occur secondarily as inflammation resolves. At present, no human UC trial has definitively shown that FMT-induced changes in Th17/Treg balance, IL-22/ILC3 activity, IgA-associated microbial selection, macrophage reprogramming, or NET formation precede and mediate clinical response. Therefore, these pathways should be regarded as biologically plausible mucosal immune mechanisms with varying levels of experimental support, rather than as validated human mediators of FMT efficacy.

Future mechanism-driven trials should combine longitudinal microbiome and metabolomic profiling with paired mucosal immune phenotyping. Useful approaches may include flow cytometry, single-cell transcriptomics, spatial transcriptomics, cytokine measurements, IgA-seq, and tissue-based imaging of neutrophil or macrophage programs. These data should be linked to clinical and endoscopic outcomes through temporal analysis and, where possible, causal mediation models. Such designs would help distinguish true mucosal immune mediators from downstream correlates of disease improvement.

## 3 Systems-level integration and causal inference

The mechanisms reviewed above—microbiota reconstitution, metabolic reprogramming, barrier repair, and immune regulation—are often discussed as separate pathways, but they are tightly interconnected within the gut ecosystem. A single microbial metabolite may simultaneously influence epithelial metabolism, barrier integrity, immune-cell differentiation, and inflammatory signaling. Butyrate, secondary bile acids, and tryptophan-derived AhR ligands therefore function less as isolated effectors than as network hubs linking microbial ecology to host responses. This systems-level property helps explain why the mechanism of FMT is difficult to reduce to a single taxon, metabolite, or signaling pathway.

### 3.1 From association to causation

A central limitation of the current mechanistic literature is the difficulty of distinguishing causal mediators from correlates of inflammation resolution. Enrichment of butyrate-producing bacteria illustrates this challenge. Although responder-associated enrichment of *Lachnospiraceae* and *Oscillospiraceae* has been reported (16), it remains unclear whether these organisms drive remission or recover secondarily as inflammation resolves and the luminal environment becomes more permissive for oxygen-sensitive anaerobes. Similar uncertainty applies to response-associated metabolic pathways, such as L-citrulline biosynthesis, which may mark a healthier microbial ecosystem rather than directly mediate therapeutic benefit (18).

Longitudinal studies have begun to challenge the simple model of “donor colonization → metabolic restoration → immune repair.” Pinto et al. (13), for example, observed that responders retained more of their original resident microbiota, suggesting that recipient ecological resilience may be as important as donor engraftment. These findings imply that FMT may act by perturbing a dysbiotic ecosystem toward a more stable, inflammation-resistant state rather than by replacing the recipient microbiota wholesale. However, most available studies lack the temporal resolution needed to prove that microbial or metabolic changes precede clinical improvement.

Future trials should therefore incorporate repeated metagenomic, metabolomic, and host-response measurements to support causal mediation analyses. Demonstrating that a putative mediator changes before clinical improvement and statistically explains part of the treatment effect would provide stronger mechanistic evidence than the cross-sectional associations that dominate the current literature.

### 3.2 Functional redundancy and ecological resilience

Another systems-level concept relevant to FMT is functional redundancy. Multiple bacterial taxa, including *Faecalibacterium prausnitzii*, *Roseburia hominis*, *Eubacterium rectale*, and members of *Clostridium* clusters, can contribute to butyrate production. Similar redundancy exists for other microbial functions, including bile acid conversion and tryptophan metabolism. Thus, therapeutic benefit may depend less on engraftment of a particular species than on restoration of missing community functions.

This principle may help explain why the same donor can produce divergent outcomes across recipients (15), and why multi-donor FMT has been associated with improved outcomes in some analyses (44). A pooled donor product may increase the probability that at least some strains or functional genes complement recipient-specific deficits. From this perspective, the search for a universal “super-donor” may be less useful than identifying functional donor–recipient complementarity. Future donor selection strategies should therefore move beyond taxonomic similarity and incorporate functional readouts, such as butyrate synthesis capacity, bile acid transformation, AhR ligand generation, and strain-level engraftment dynamics.

### 3.3 Future mechanistic priorities

In this review, recipient ecological receptivity refers to the capacity of the recipient intestinal ecosystem to permit donor-derived or functionally beneficial microbial features to persist and exert host effects. It may be indexed by baseline microbial diversity, retention of recipient-native taxa, depletion of pathobionts, inflammatory burden, mucosal barrier status, metabolic deficits, and donor–recipient functional complementarity.

Several questions remain particularly important at the mechanistic–clinical interface. What determines recipient ecological receptivity, and can it be enhanced by diet, mucosal protection, or targeted preconditioning? Is sustained colonization by live donor bacteria required, or can postbiotics, sterile filtrates, or extracellular vesicles reproduce key therapeutic effects? What governs donor-strain durability and relapse after initial response? Finally, what are the safe and effective concentration ranges for microbiota-derived metabolites such as butyrate and secondary bile acids?

Addressing these questions will require mechanism-driven FMT trials that combine dense longitudinal sampling, functional microbiome profiling, metabolomics, and host mucosal readouts. Such studies would help move the field from descriptive association toward testable causal models and provide a stronger foundation for precision microbiota therapy. The main mechanisms and evidence gaps of FMT in UC are summarized in **Table 1**.

**Table 1:** Main proposed mechanisms, evidence levels, and evidence gaps of fecal microbiota transplantation in ulcerative colitis.

Mechanism	Key mediators	Reported effect	Evidence level	Evidence gap
Microbiota restoration and ecological resilience	Donor/resident taxa; functional capacity (13–15, 44)	Associated with microbial ecological changes	Human longitudinal and clinical cohort evidence	Requirement for durable engraftment remains unclear
SCFA metabolism	Butyrate producers; SCFAs (16, 17, 19)	Associated with barrier and immune regulation	Human associative evidence supported by preclinical mechanistic data	Causality remains uncertain
Bile acid remodeling	Secondary bile acids; FXR/TGR5 (20–22)	Linked to anti-inflammatory signaling	Human associative evidence and mechanistic inference	Safe and effective concentration range is undefined
Tryptophan–AhR axis	Indoles; AhR; IL-22 (23–25)	Linked to epithelial and immune regulation	Human associative evidence with animal causal support	Human causal evidence remains limited
Barrier repair	ZO-1; occludin; MUC2 (26–31)	Associated with improved epithelial barrier markers	Human transcriptomic evidence and animal protein-level evidence	Functional validation in humans is lacking
Immune regulation	Th17/Treg; IL-10 (31, 35–37)	Associated with mucosal immune rebalance	Human associative evidence with gnotobiotic and preclinical support	Human causal evidence remains limited
Ferroptosis/EVs	GPX4; microbial EVs (32–34, 45, 46)	Suggested by preclinical studies	Mainly preclinical evidence	Clinical relevance in UC remains unproven

UC, ulcerative colitis; FMT, fecal microbiota transplantation; SCFA, short-chain fatty acid; AhR, aryl hydrocarbon receptor; EVs, extracellular vesicles; GPX4, glutathione peroxidase 4; Treg, regulatory T cell.

Evidence-level definitions: Evidence-level terms are used descriptively rather than as a formal evidence-grading system. “Human associative evidence” refers to clinical, microbiome, metabolomic, transcriptomic, or immunologic associations observed in patients without causal perturbation. “Human longitudinal and clinical cohort evidence” refers to repeated sampling, longitudinal microbiome analysis, or predictive cohort analyses in human FMT studies. “Human transcriptomic evidence” refers to mucosal gene-expression data from human samples without direct functional validation. “Animal/gnotobiotic mechanistic support” refers to experimental colitis, germ-free, or defined-colonization models in which strains, metabolites, or immune pathways are functionally tested. “Preclinical mechanistic data” refers to animal or *in vitro* studies supporting pathway-level plausibility. “Mainly preclinical evidence” indicates that support is largely derived from animal or *in vitro* studies, with limited or no direct human UC validation. Composite phrases in the Evidence level column indicate that more than one type of evidence supports the same mechanism.

## 4 Clinical evidence and therapeutic boundaries

### 4.1 Systematic reviews and meta-analyses

By early 2026, more than a dozen meta-analyses had systematically appraised FMT efficacy in UC. Across randomized trials, pooled analyses generally suggest that FMT improves clinical and endoscopic remission relative to placebo, with reported effect estimates broadly favoring FMT (47, 48). These summary estimates, while superficially robust, obscure substantial inter-study heterogeneity in donor number, preparation method, route of administration, and patient population—heterogeneity that directly undermines the clinical utility of a single pooled effect size. Network meta-analysis further suggests that route of delivery may be a major determinant of efficacy, with lower gastrointestinal or combined approaches performing better than upper gastrointestinal delivery alone (49). These findings suggest that the statement “FMT is effective” has limited clinical meaning unless formulation, delivery route, regimen, and patient population are specified. Across trials included in meta-analyses, protocols differed in donor number, route of administration, dosing intensity, pretreatment antibiotics, and co-interventions such as diet, which limits the interpretability of pooled effect estimates.

Safety syntheses face analogous limitations. Available meta-analytic evidence does not show a significant excess of adverse events with FMT compared with placebo, but most trials excluded patients with severe disease and followed participants for only 8–12 weeks (50). The rarity of serious adverse events renders confidence intervals wide, and the short follow-up precludes detection of late-emerging complications. Moreover, the methodological quality of existing systematic reviews is variable, with certainty of evidence ranging widely across outcomes (51). Thus, although a signal of efficacy is present, the quality and heterogeneity of available syntheses remain insufficient to support FMT as a standard-of-care recommendation in clinical guidelines.

### 4.2 Key randomized controlled trials

#### 4.2.1 Positive trials

The LOTUS trial (52) was the first double-blind RCT of oral lyophilized FMT. At 8 weeks, the combined endpoint was met by 53% of the FMT group versus 15% of the placebo group; the four patients who continued FMT during the maintenance phase all remained in remission at week 56. Generalizability is constrained, however, by the small sample (n = 35), early termination due to the COVID-19 pandemic, universal antibiotic preconditioning—which precludes separation of the antibiotic effect from the FMT effect—and the tiny maintenance cohort. The 100% maintenance rate in four patients, while encouraging, cannot be distinguished from a selection effect in a sample of this size. The FMT-AID trial (53) bundled multi-donor FMT with an anti-inflammatory diet; deep remission (combined clinical and endoscopic) was achieved in 36.4% versus 8.7% at 8 weeks, with diet-alone maintenance showing sustained benefit at 48 weeks. The open-label design and the inseparable dietary co-intervention leave the relative contribution of FMT indeterminate, although the sustained benefit in the diet-alone maintenance phase suggests that dietary modification may be an underappreciated contributor to long-term outcomes. The multi-donor intensive regimen of Paramsothy et al. (54)—an initial colonoscopic infusion followed by enemas 5 days per week for 8 weeks—produced steroid-free clinical remission with endoscopic remission or response in 27% versus 8% (placebo), but its operational burden (initial colonoscopy plus 40 self-administered enemas) and the subsequent recognition that not all patients require such intensity have limited its real-world applicability. These three trials collectively established proof-of-concept but left unresolved the questions of optimal dosing intensity, the independent contribution of FMT versus co-interventions, and the durability of response beyond 12 months.

#### 4.2.2 Negative and boundary-defining trials

The RESTORE-UC trial (2025) is one of the most informative negative RCTs to date, not merely because it failed to meet its primary endpoint, but because its design helps clarify therapeutic boundaries of FMT that positive trials could not fully define. Despite multidimensional donor screening—high cell counts (>1.75 × 10¹¹ cells/g), specific enterotypes, exclusion of Bacteroides 2 enterotype, and low abundance of Fusobacterium, Escherichia/Shigella, and Veillonella—and anaerobic preparation, the allogeneic FMT arm achieved corticosteroid-free clinical remission in only 10.0% at 8 weeks, not significantly different from the 13.9% observed with autologous FMT (P = 0.72); the trial was halted for futility after 66% of planned inclusions (55).

The reasons for failure warrant close analysis because they may help clarify settings in which FMT is less likely to be effective. First, the enrolled population had moderate-to-severe disease (total Mayo 4–10), and the baseline inflammatory burden likely exceeded the reversible range for a microbiota-alone intervention. This suggests that the therapeutic window for FMT monotherapy may be narrower in patients with higher inflammatory burden, and that beyond a certain inflammatory threshold, concurrent anti-inflammatory pharmacotherapy may be required. Second, the comparable response rates in the allogeneic and autologous arms (10.0% vs 13.9%, P = 0.72), which were not statistically different, suggest that non-specific procedural effects or a placebo response may, in part, account for the outcomes in both groups, rather than indicating a genuine therapeutic effect of allogeneic FMT. The original investigators consequently recommended using sterilized autologous solutions as a more rigorous sham control in future trials (55). While the possibility of intrinsic anti-inflammatory constituents in autologous FMT cannot be entirely excluded, the data from this halted trial are insufficient to support this hypothesis. Third, four FMT administrations may have been insufficient to establish durable donor engraftment in a hostile inflammatory environment.

The STOP-Colitis trial (56) compared colonoscopic with nasogastric FMT delivery. Clinical response was 75% in the colonoscopy arm versus 25% in the nasogastric arm. Although this was an open-label feasibility study of 30 patients with a high dropout rate in the nasogastric group (50% vs 14%), the magnitude of the signal supports further prioritization of lower gastrointestinal delivery in future trials, although confirmation in larger cohorts is needed. The mechanistic inference—that nasogastric delivery fails to convey sufficient numbers of viable anaerobic organisms to the colonic target—is consistent with the known oxygen sensitivity of key donor taxa and supports the principle that the delivery route must match the ecological requirements of the transplanted community.

Taken together, these boundary-defining trials identify a fundamental constraint that positive trials could not have revealed. The capacity of donor microbiota to occupy niches in the recipient gut depends on the receptivity of the host microenvironment and the reversibility window determined by disease stage. When inflammation surpasses the threshold that microbiota modulation can surmount (moderate-to-severe UC), or when the delivery route fails to convey anaerobic organisms to the colonic target (upper gastrointestinal administration), the ecological reconstruction necessary for therapeutic benefit may be difficult to achieve. This limitation does not invalidate FMT; rather, it delineates its clinical coordinates: efficacy may depend strongly on disease severity, delivery route, and recipient ecological receptivity. Recognizing these potential boundaries may help inform more rational trial design and clinical decision-making. RESTORE-UC, in particular, implies that even a well-characterized “optimal” donor cannot compensate for a non-receptive host environment, shifting the investigative emphasis from the hunt for super-donors toward the understanding and improving recipient ecological receptivity—a concept introduced in Section 2.1. Key randomized controlled trials evaluating FMT in UC are summarized in **Table 2**.

**Table 2:** Key clinical evidence of FMT in UC.

Study	Population	FMT regimen	Main result	Interpretation
Paramsothy et al., 2017 (54)	Active UC	Multi-donor FMT; colonoscopy + intensive enemas	27% vs 8% achieved the primary endpoint	Supports efficacy of intensive multi-donor FMT in selected patients
Haifer et al./LOTUS, 2022 (52)	Active UC	Oral lyophilized FMT capsules after antibiotics	53% vs 15% achieved the primary endpoint	Suggests potential efficacy of oral FMT, but sample size was small
Kedia et al./FMT-AID, 2022 (53)	Mild-to-moderate UC	Multi-donor FMT + anti-inflammatory diet	36.4% vs 8.7% achieved deep remission	Suggests benefit of a diet-assisted strategy; FMT-specific effect is difficult to isolate
Caenepeel et al./RESTORE-UC, 2025 (55)	Moderate-to-severe UC	Single-donor anaerobic allogeneic FMT	10.0% vs 13.9%; stopped for futility	Did not show benefit in this moderate-to-severe population
Quraishi et al./STOP-Colitis, 2026 (56)	Active UC	Colonic vs nasogastric delivery	75% vs 25% clinical response	Suggests route-dependent differences; larger confirmatory trials are needed

UC, ulcerative colitis; FMT, fecal microbiota transplantation. Additional randomized controlled trials of FMT in UC are summarized in **Supplementary Table 1**.

### 4.3 Administration route and regimen optimization

Current evidence increasingly supports lower gastrointestinal delivery, although optimal delivery strategies still require further refinement (49, 56). Some analyses have suggested better outcomes with multi-donor (MDN) than single-donor (SDN) FMT (RR, 2.31) (44), although Chapon et al. (49) found no significant difference in their network meta-analysis, suggesting that regimen intensity and patient characteristics may confound the apparent effect of donor number. Higher fecal doses (>275 g total feces) are associated with greater efficacy (57). Lower gastrointestinal delivery should therefore be viewed as better supported by current evidence, rather than as definitively established as the universally preferred route.

**Figure 2:**
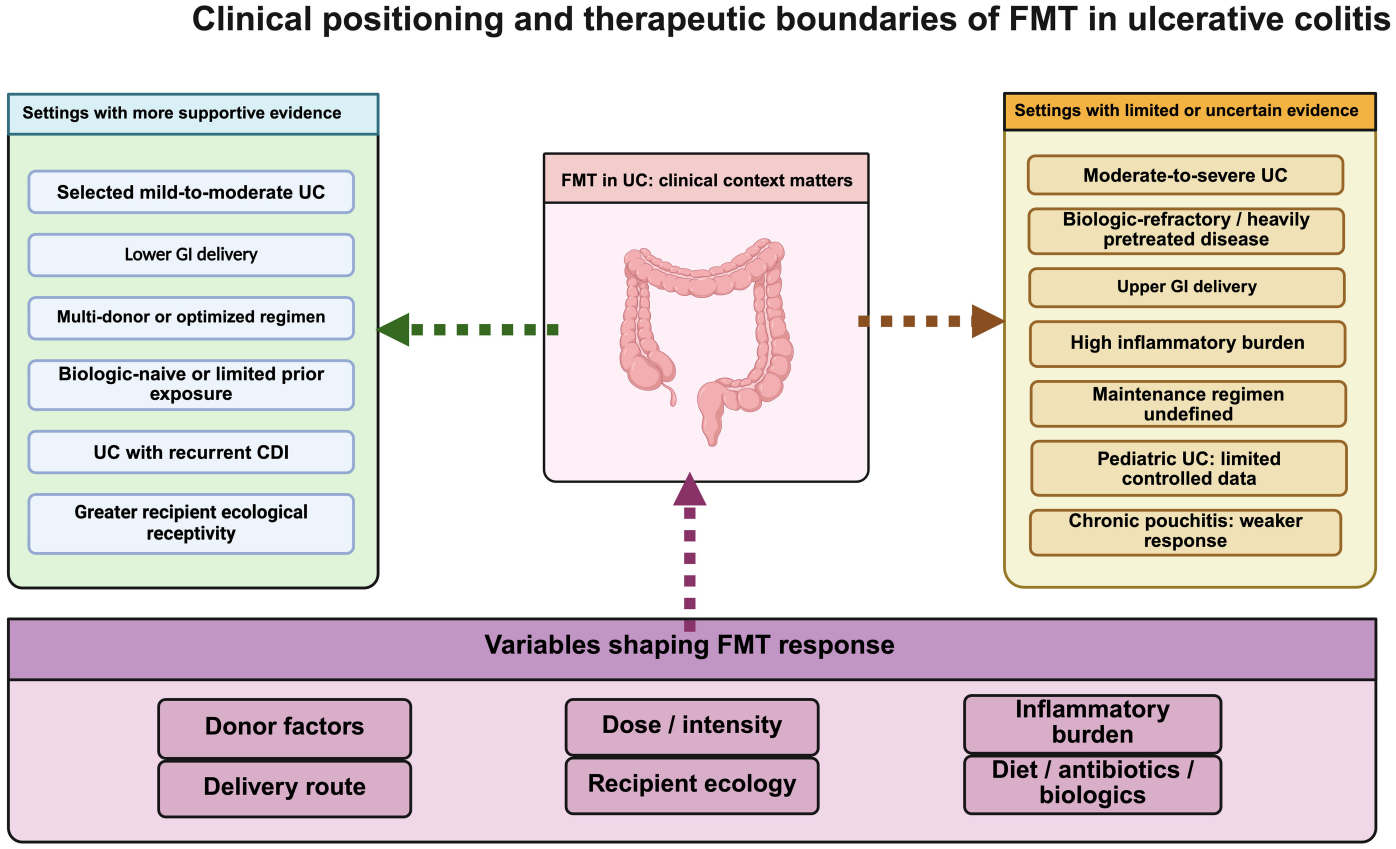
Clinical positioning and therapeutic boundaries of fecal microbiota transplantation in ulcerative colitis. Current evidence suggests that FMT outcomes vary according to disease severity, delivery route, donor composition, treatment intensity, recipient ecological receptivity, and co-interventions. Evidence is more supportive in selected patients with mild-to-moderate UC treated with lower gastrointestinal delivery and optimized regimens, whereas benefit remains limited or uncertain in moderate-to-severe, biologic-refractory, pediatric, maintenance, and pouchitis settings. These categories are intended as a framework for clinical interpretation and trial design rather than definitive treatment recommendations. Created with BioRender.com under a publication license. Created in BioRender under a publication license. Source: https://BioRender.com/f9i7t9y.

Maintenance therapy constitutes one of the most consequential evidence gaps in the field. Lahtinen et al. (58) reported that a single FMT did not reduce 12-month relapse rates, whereas Li et al. (59) found in a real-world setting that a second FMT extended median time to relapse from 120 to 182.5 days. The discrepancy between these findings is consistent with a model in which repeated dosing is required to compensate for the natural decay of donor strains over time. The decay kinetics themselves, however, have never been systematically tracked. Without an understanding of the rate and determinants of donor strain loss, the optimal interval for re-treatment cannot be rationally determined, nor can patients at risk of early relapse be prospectively identified. Until maintenance strategies are rigorously tested in dedicated RCTs with adequate follow-up, FMT will remain confined to induction therapy, and its long-term role in UC management will remain undefined. These clinical contexts and variables shaping FMT response are summarized in **Figure 2**.

### 4.4 Special subgroups

#### 4.4.1 Refractory and biologics-exposed UC

Nearly all existing RCTs excluded patients with recent biologic exposure; evidence for this population derives exclusively from observational studies and underpowered subgroup analyses. A meta-analysis of six refractory-UC RCT subgroups found no significant benefit on clinical or endoscopic endpoints, albeit with variable definitions of refractoriness and small sample sizes (60).

A non-randomized, exploratory study of patients with refractory IBD reported a potential clinical benefit when FMT was combined with infliximab (clinical response in 6 of 9 patients); additive effects on the gut microbiome and metabolome, including restoration of α-diversity and normalization of bile acid and amino acid profiles, were observed in this small combination therapy subgroup (61). A pilot study of FMT plus vedolizumab was terminated early because of recruitment difficulties, without safety concerns (62).

Combination strategies are conceptually appealing—resting on the rationale that immunosuppression may facilitate donor engraftment while microbiota restoration may reduce immunogenicity—but remain at an early, largely anecdotal stage of evidence. The absence of RCT data in this population is particularly consequential given that biologic-refractory patients represent precisely the group for whom novel therapeutic options are most urgently needed.

#### 4.4.2 UC with recurrent C. *difficile* infection

This subgroup has the strongest evidentiary foundation for FMT. Observational data suggest that FMT is effective for recurrent CDI in patients with concurrent UC, with reported CDI cure rates improving after repeat procedures and UC worsening appearing uncommon in available cohorts (63, 64). For UC patients with recurrent CDI, FMT may therefore be considered early, as the dual benefit—CDI eradication and potential UC improvement—is supported by consistent observational evidence, and the risk-benefit ratio compares favorably with prolonged antibiotic strategies.

#### 4.4.3 Pediatric UC

A meta-analysis by Hsu et al. (65) estimated clinical response and remission rates of 58.8% and 64.7%, respectively, within four weeks of FMT. No included study, however, was an RCT, and sample sizes were uniformly small. The largest pediatric cohort (74 children) with 5-year follow-up detected no safety signals (66), though five years is plainly insufficient to exclude late-emerging neoplastic or autoimmune sequelae. Given the particularly long lifetime risk horizon in pediatric patients, the absence of controlled data should temper enthusiasm for FMT in this population outside of clinical trials. Informed consent should therefore involve careful discussion with guardians and, when appropriate, assent from the child, and pediatric FMT should preferably be restricted to controlled studies or situations with a strong clinical rationale.

#### 4.4.4 Pouchitis

FMT performs substantially worse in chronic pouchitis than in UC, a discrepancy that provides indirect insight into the ecological requirements for FMT efficacy. A meta-analysis reported a pooled remission rate of 15% (95% CI, 0%–29%), a clinical response rate of 33%, and a relapse rate of 36% (67). An RCT found no prolongation of relapse-free survival at 52 weeks (68). The pouch, which more closely resembles ileal than colonic ecology—with facultative anaerobes predominating and obligate anaerobes colonizing poorly—likely requires dedicated protocols that account for its distinct microbial and immunologic environment, rather than simple extrapolation from colonic UC protocols.

### 4.5 Safety, adverse events, and regulatory landscape

Short-term adverse events are predominantly mild and self-limited. Washed preparation reduces the febrile reaction rate from approximately 19.4% to 2.7% (69), although whether the washing process also depletes immunomodulatory soluble factors or EVs is unresolved—a concern sharpened by the finding that cell-free EVs outperform whole FMT in an experimental colitis model (46). The impact of persistent, albeit “mild,” symptoms such as bloating and diarrhea on quality of life is frequently underappreciated in RCT reporting and deserves systematic assessment in future trials using validated patient-reported outcome measures.

The regulatory landscape was permanently altered by the report by DeFilipp et al. (70) of two cases of ESBL-producing *E. coli* bacteremia transmitted by FMT, one of which was fatal. This sentinel event transformed donor screening from a routine procedure into the central cost driver of FMT programs, with eligibility rates now as low as 3%–25% (71). Consequently, FMT has been transformed from an inexpensive intervention into a costly, logistically demanding procedure—a shift with direct implications for health equity, particularly as the UC burden increases in resource-constrained settings. The second ROME consensus (2025) proposed a standardization framework (72), but it is more accurately described as a template for future RCTs than a mature clinical guideline, reflecting the underlying fragility of the evidence base on which recommendations must rest.

Published safety reports have emphasized that infectious risks after FMT are not theoretical. Transmission of drug-resistant Escherichia coli has been reported after FMT, including ESBL-producing E. coli bacteremia with one fatal outcome (70). Viral transmission also requires attention. During the COVID-19 pandemic, international expert recommendations emphasized updated donor screening, exclusion of donors with relevant symptoms or exposure history, consideration of stool-based molecular testing for SARS-CoV-2 when feasible, and risk-specific informed consent (73, 74). These reports support dynamic donor-screening protocols that are updated in response to emerging bacterial, viral, and antimicrobial-resistance threats.

Long-term safety beyond the 5-year horizon remains unaddressed and constitutes perhaps the most significant uncertainty for clinical adoption. Follow-up of 25.5 months (median) by Li et al. (59) and 5-year pediatric data from Zou et al. (66) revealed no signals of malignancy or autoimmunity, but these intervals are far too brief given typical latencies for solid tumors and many immune-mediated conditions. FMT introduces an entire allogeneic microbiota—replete with immunostimulatory components including lipopolysaccharide, flagellin, and CpG DNA—into the recipient gut. Whether this exposure durably resets host immune thresholds, and whether such reprogramming could manifest as autoimmune or neoplastic disease a decade or more later, is entirely uncharted territory. Answers will require coordinated international registries with follow-up extending well beyond the conventional trial horizon—an infrastructure that currently does not exist.

A further, often overlooked, safety dimension concerns the long-term metabolic consequences of FMT. Post-FMT elevations in butyrate and secondary bile acids are well documented, but their concentration-response relationships are unexplored. Excess butyrate may suppress epithelial stem cell proliferation; sustained high concentrations of secondary bile acids are potentially genotoxic. That these metabolites occupy a therapeutic “double-edged sword”—anti-inflammatory at physiological levels, potentially harmful at supraphysiological ones—has not been systematically considered in any FMT trial, and no target concentration range has been defined for any metabolite. Incorporating metabolomic monitoring into long-term safety follow-up would address this gap while also informing the therapeutic window for metabolite-based therapies.

### 4.6 FMT and biologics

The coexistence of FMT and biologic therapies in UC raises an unresolved clinical question: should these mechanistically distinct strategies be used competitively, sequentially, or in combination? No head-to-head RCT has directly compared FMT with biologic or small-molecule therapies. An indirect network meta-analysis by Vuyyuru et al. (75) found no statistically significant difference between FMT and several targeted agents for induction of clinical remission, but the comparison was highly indirect because of differences in eligibility criteria, endpoints, and treatment regimens. This finding should therefore be interpreted as an absence of definitive comparative evidence rather than evidence of therapeutic equivalence.

Combination therapy remains hypothesis-generating. Small pilot studies have explored FMT combined with infliximab or vedolizumab (61, 62), but none included an adequately powered monotherapy comparator. Two theoretical rationales support further investigation: microbiota restoration may reduce immunogenicity and improve biologic durability, whereas biologic-induced suppression of mucosal inflammation may improve donor engraftment. One possibility is that biologic-induced suppression of mucosal inflammation may create a more permissive ecological niche for donor engraftment by reducing epithelial injury, inflammatory oxygenation, antimicrobial pressure, and diarrhea-associated washout. However, the optimal timing remains unknown. A biologic-first strategy might improve ecological receptivity by reducing inflammation, whereas an FMT-first strategy might reshape the microbiota before immune-targeted treatment. These possibilities have not been prospectively compared.

Current evidence is therefore insufficient to recommend FMT as a substitute for biologic therapy or to define its optimal sequence relative to biologics. The low response observed in RESTORE-UC (55) suggests that advanced inflammatory burden and prior treatment exposure may limit FMT responsiveness, but this does not establish that FMT should be used before biologics. Rather, an earlier, biomarker-selected use of FMT before biologic failure deserves prospective evaluation, preferably in trials of biologic-naïve mild-to-moderate UC. Until such data are available, FMT–biologic integration should remain investigational and be tested through biomarker-stratified, sequential-strategy or combination trials.

### 4.7 Health economic considerations

From a health economic perspective, adding FMT to standard care for mild-to-moderate UC has been modeled as cost-effective over a 10-year horizon, with an incremental cost-effectiveness ratio below the conventional $50,000/QALY threshold (76). In this model, cost savings were primarily driven by FMT-induced remission reducing the need for subsequent costly treatment escalation. If FMT were shown to induce durable long-term remission and reduce lifetime biologic exposure in a meaningful subset of patients, its economic attractiveness could increase further. This scenario, however, remains contingent on resolving the durability question identified in Section 4.3 and would require dedicated long-term pharmacoeconomic modeling.

## 5 Toward precision microbiota therapeutics

**Figure 3:**
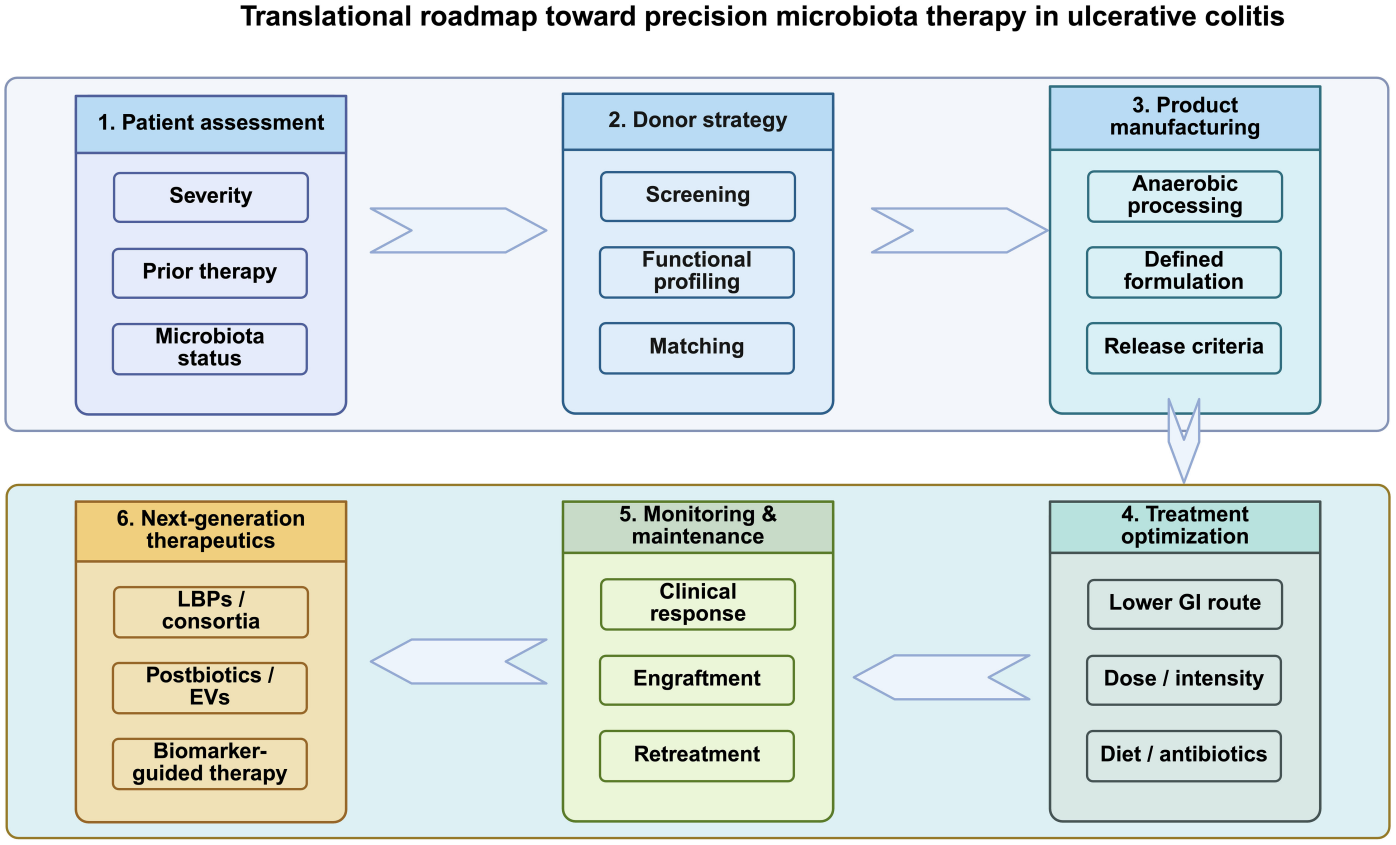
Translational roadmap toward precision microbiota therapy in ulcerative colitis. The transition from empirical FMT to precision microbiota therapy requires patient assessment, donor functional profiling, standardized product manufacturing, treatment optimization, longitudinal monitoring, and development of next-generation microbiota-based therapeutics. Major implementation barriers include limited prospective validation, lack of standardized release criteria, uncertain long-term safety, undefined maintenance regimens, and regulatory and cost constraints. Created with BioRender.com under a publication license. Created in BioRender under a publication license. Source: https://BioRender.com/gs7xeia

The marked inter-individual variability in FMT response raises a set of interlocking questions that define the frontier of the field. Why does efficacy differ so dramatically between apparently similar patients? Can responders be identified before treatment? Can donor–recipient pairing be optimized, or can donors be bypassed altogether? This section examines the translational strategies that address these questions, focusing on approaches with near-term clinical feasibility while acknowledging the implementation barriers that stand between precision science and routine practice. We argue that the path forward lies not in any single innovation, but in the systematic integration of donor optimization, manufacturing rigor, matching algorithms, rational combination therapy, and pragmatic trial designs that reflect real-world constraints. A translational roadmap toward precision microbiota therapy in UC is shown in **Figure 3**.

### 5.1 Donor screening and the super-donor concept

Donor eligibility rates of 3%–25% (71) reflect the stringency of modern screening protocols. Yet an “eligible” donor is far from an “effective” one. In the LOTUS trial, two donors screened under identical criteria produced clinical plus endoscopic remission in 100% versus 36% of recipients (15), a divergence that conventional screening parameters could not explain. “Super-donor” microbiota are characterized by high α-diversity, species evenness, enrichment of Bacteroidota, and temporal stability. Although high diversity, taxonomic richness, anaerobe enrichment, and favorable metabolic capacity have been proposed as desirable donor features, none has consistently predicted response across trials. Crucially, these traits are not absolute donor properties; they are relational attributes whose expression depends on the recipient context. The LOTUS data, in which the same donor appeared highly effective in some recipients but less effective in others, provide important empirical support for this relational model. There is, on current evidence, no universal donor—a conclusion with direct implications for donor selection strategies, which should prioritize functional profiling of donor–recipient pairs over the identification of intrinsically superior donors.

Multi-donor FMT has been associated with better outcomes than single-donor FMT in some analyses (44), and one possible explanation is statistical: pooling donors increases the combinatorial diversity of functional genes, raising the probability that at least some donor-derived strains will fill recipient-specific functional deficits. This logic immediately raises subsidiary questions that remain unaddressed: what is the optimal mixing ratio, and can donor combinations be tailored to the recipient’s known microbiota deficiencies? The current practice of equal-proportion mixing may be suboptimal, although comparative data are lacking. A longer-term vision involves donor strain banks from which prescription-based consortia could be assembled according to the functional gaps identified in a given recipient—an approach that presupposes large-scale strain isolation, functional annotation, and a manufacturing infrastructure that does not yet exist. The feasibility of this vision will depend less on scientific discovery than on investment in the biobanking and characterization infrastructure required to make functional donor matching a clinical reality.

### 5.2 Manufacturing and quality control

Donor screening is merely the first node in a quality chain that extends from defecation to infusion. The viability, composition, and function of the microbiota can change dramatically at each step, yet manufacturing standardization has received far less investigative attention than donor selection. This asymmetry—intensive focus on donor characteristics with comparatively minimal attention to what happens to the microbiota between donation and delivery—represents a significant blind spot in current FMT research.

Oxygen exposure is a critical and frequently uncontrolled variable. The gut microbiota is dominated by obligate anaerobes, but most FMT preparation is performed under ambient air. Bénard et al. (77) demonstrated that anaerobic processing substantially improves the survival of butyrate-producing genera such as *Faecalibacterium* and *Blautia in vitro*; Shimizu et al. (78) reported significantly higher viable counts of the *Clostridium* coccoides group and B. fragilis group under anaerobic conditions in ex vivo preparations. These laboratory-based findings have led to the hypothesis that the common practice of documenting “frozen versus fresh” comparisons without reporting the extent of oxygen exposure during preparation may contribute to the unexplained heterogeneity of clinical trial results. However, direct evidence linking the degree of oxygen exposure during FMT preparation to clinical outcomes in UC is currently lacking. Prospective trials that systematically document and control for oxygen exposure are needed to test this hypothesis. Future trials should, at minimum, report oxygen exposure metrics as part of their manufacturing quality control documentation.

Washed microbiota transplantation (WMT) has been advanced as a safety-enhancing refinement. Automated washing reduces the febrile reaction rate from 19.4% to 2.7% without apparent loss of efficacy (69). As noted in Section 4.5, whether the washing process also depletes immunomodulatory soluble factors or EVs remains an unresolved concern. No head-to-head efficacy comparison of WMT with conventional FMT has been performed, so the safety advantage alone does not yet justify universal adoption. This equipoise should be resolved through a dedicated non-inferiority trial before WMT is widely implemented.

The standardization of frozen and lyophilized formulations is essential for the transition from artisanal to pharmaceutical-grade FMT. Freezing at −80 °C preserves microbiota composition for months, and lyophilization extends shelf life while enabling oral capsule delivery. Meta-analytic data from recurrent CDI suggest equivalent efficacy of capsule and fresh FMT (79), but extrapolation to UC is problematic: UC involves more complex host–microbiota–immune interactions, and whether the selective loss of freeze-sensitive species—*F. prausnitzii* prominent among them—disproportionately compromises efficacy in UC has not been studied. Given the centrality of butyrate producers to the hypothesized mechanism of action, this is not merely a technical question but a mechanistic one that should be addressed through head-to-head comparisons of fresh versus lyophilized FMT in UC-specific RCTs.

The most fundamental manufacturing deficit, however, is the absence of positive release criteria. Current product release is governed almost entirely by negative donor screening results. No consensus exists on the minimum viable bacterial count, the requisite representation of key species, or the functional metabolic activity that an FMT dose should possess. Zhao et al. (57) identified a fecal weight >275 g as more effective, but the microbial activity within “275 g of feces” may vary substantially, and fecal weight remains a crude proxy for the biologically active fraction of the transplant. An ideal quality system would incorporate flow-cytometric total viable counts, qPCR quantification of sentinel taxa, and functional determination of butyrate production capacity. Serum metabolic profiling has also been explored for predicting FMT efficacy in patients with extensive UC (80). Such a system is currently too costly for routine deployment, but its absence means that the true “active ingredient” of FMT may vary considerably between studies, between centers, and even between batches from the same center. This variability may contribute substantially to the heterogeneity of clinical trial results and must be addressed before meaningful inter-trial comparisons can be drawn. The field would benefit from a consensus-driven effort, analogous to the minimum information guidelines developed for microbiome studies, to define the essential quality control parameters for FMT product release.

### 5.3 Donor–recipient matching

A systematic review of donor and recipient predictive biomarkers has cataloged the range of candidate predictors currently under investigation (81). Three preliminary matching models have been described, each using a different methodological approach: an analytic hierarchy process model based on 16S datasets, a random forest classifier incorporating three bacterial genera, and a clinical nomogram using age, disease extent, and endoscopic severity (82–84). Although these models reported encouraging retrospective performance, none has yet been prospectively validated, and no trial has shown that algorithm-guided donor selection improves outcomes over empirical matching. This gap between retrospective promise and prospective validation remains an important barrier to clinical implementation of precision matching.

A deeper conceptual issue is that these models capture correlation, not causation. High baseline *Candida* abundance is associated with FMT response (23), but is *Candida* a mechanistic driver or merely a correlate of a healthier ecosystem? Unless this distinction is made—through experimental perturbation, not observational association—matching donors on such features risks simply selecting patients with a higher *a priori* probability of response, irrespective of the donor. Behling et al. (85) showed in two independent RCTs that greater donor–recipient microbiota similarity was associated with superior engraftment in UC but the reverse in obesity, demonstrating that matching rules are disease-specific and cannot be generalized across indications. The field may also have over-invested in “microbiota similarity” at the expense of “functional complementarity.” The metabolic functions missing in a given recipient—butyrate synthesis, bile acid conversion, AhR ligand generation—may be restored by specific donor strains that are taxonomically dissimilar to the recipient’s resident microbiota. Redefining the matching distance in metagenomic functional space, rather than taxonomic space, represents an important conceptual advance that now requires prospective validation. A pragmatic next step would be a randomized trial comparing functional-gene-based donor matching with standard empirical donor allocation, using donor strain engraftment at week 4 as a biologically proximal endpoint before larger trials powered for clinical outcomes are undertaken.

### 5.4 Combination therapy

#### 5.4.1 Antibiotic preconditioning

The A-FMT regimen (amoxicillin, fosfomycin, metronidazole) produced 4-week clinical response and remission rates of 62.9% and 36.1%, respectively, in a cohort of 97 UC patients (86). Whether efficacy would be lower without antibiotic preconditioning is unknown, because the LOTUS regimen administered oral lyophilized FMT after antibiotic pretreatment and lacked a no-antibiotic comparator. This limitation should not be generalized to RESTORE-UC, in which universal recipient antibiotic preconditioning was not part of the trial design; antibiotics appeared as part of donor exclusion criteria, and recipient antibiotic pretreatment was mentioned only as a potential consideration for future studies. The broader evidence gap is therefore whether antibiotic preconditioning itself improves engraftment or efficacy when directly compared with no preconditioning. Broad-spectrum antibiotics may clear beneficial bacteria along with pathogenic bacteria, creating vacant niches that opportunistic colonizers may occupy. The ideal preconditioning strategy would approximate precision weeding—selectively eliminating pathobionts while sparing beneficial commensals—but currently available antibiotics remain blunt instruments. Until more selective preconditioning regimens are developed and tested against no-preconditioning controls, the routine use of antibiotics before FMT for UC should be regarded as investigational rather than standard practice.

#### 5.4.2 Dietary intervention

Diet is conceptually attractive because it shapes both donor and recipient microbiota. The FMT-AID trial, however, bundled diet with FMT, precluding isolation of the independent FMT effect (53). Despite this limitation, the trial’s sustained benefit in the diet-alone maintenance phase provides a strong rationale for further investigation of dietary strategies.

Leibovitzh et al. (87) demonstrated, in a *post-hoc* analysis of the CRAFT UC trial, that a 14-day donor conditioning diet depleted sulfur-containing amino acid pathways (L-methionine and L-cysteine biosynthesis) in the donor microbiota; the recipient Eubacterium sp. AF228LB increased post-FMT and correlated inversely with fecal calprotectin (rho = −0.52, p = 0.035). This study is noteworthy because it provides preliminary, proof-of-concept evidence that donor dietary conditioning may influence the taxonomic and functional properties of the transplanted microbiota, which in turn may affect recipient outcomes. However, as the authors acknowledge, the study cannot disentangle whether the favorable microbial changes are donor-derived (due to donor dietary conditioning) or recipient-derived (due to the post-FMT UC Exclusion Diet), and the small sample size (n = 10 per group) limits generalizability.

Adherence and standardization pose formidable obstacles to scalability, and whether dietary effects on the microbiota are sufficiently durable to influence FMT outcomes in a clinical setting remains to be demonstrated. Further studies designed to isolate the independent contributions of donor conditioning and recipient diet are needed to translate this proof-of-concept into actionable clinical protocols.

#### 5.4.3 FMT combined with biologics

The theoretical case for combining FMT with biologics—and the unresolved questions regarding optimal sequencing—has been discussed in detail in Section 4.6. To briefly recapitulate: mechanistic complementarity provides a compelling rationale (microbiota restoration may reduce immunogenicity, while immunosuppression may facilitate engraftment), but the incremental benefit over either modality alone remains entirely unquantified. Future trials should compare FMT monotherapy, biologic monotherapy, and combination therapy, while incorporating biomarker analyses to determine whether specific patient subsets derive additional benefit from combined treatment.

### 5.5 Next-generation microbiota therapies

Live biotherapeutic products (LBPs) seek to replace whole-microbiota transplantation with defined, reproducible strain consortia. Their advantages—batch-to-batch consistency, exclusion of unknown pathogens, regulatory tractability—are offset by an unresolved question of fundamental importance: can a small consortium, or even a single strain, recapitulate the metabolic network redundancy and ecological resilience of an intact microbiota? The answer has direct implications for the scalability of LBPs; if functional redundancy is essential for sustained therapeutic effect, consortia may need to be considerably more complex than those currently in development. Engineered probiotics represent a further iteration, in which bacteria are genetically modified to sense inflammatory cues and deliver therapeutic molecules *in situ* (88, 89).

Postbiotics and sterile fecal filtrates pursue a live-bacteria-free strategy that, if successful, would represent a genuine paradigm shift. The FRESCO trial is evaluating whether a <0.2 μm sterile multi-donor stool filtrate retains clinical activity in UC (90). Should filtrates prove effective, the implication would be that the therapeutically active constituents of FMT are soluble molecules or viral particles rather than viable bacteria—a finding that would fundamentally alter the risk-benefit calculus by eliminating the infectious and engraftment-related risks of whole-microbiota transplantation. Zu et al. (46) have already shown that microbiota-derived EVs can outperform whole FMT in an animal model, providing preclinical proof-of-concept for this approach. These findings remain speculative for conventional FMT in UC because most evidence derives from engineered or experimentally conditioned EV preparations rather than clinical FMT products. Postbiotics offer freedom from infectious risk and easier standardization, but their mechanisms of action are incompletely understood, optimal dosing is undefined, and the possibility that chronic exogenous metabolite supplementation might suppress endogenous microbial functions has not been explored—a concern that applies equally to any sustained metabolite-based intervention.

It is unlikely that any single next-generation modality will replace all forms of FMT. A more cautious and clinically realistic prospect is stratified development across different clinical contexts, provided that efficacy and safety are validated in dedicated trials. For selected patients with mild-to-moderate, treatment-naïve UC, defined LBPs or postbiotic approaches may eventually be explored as lower-risk microbiota-based strategies, but they should not yet be viewed as established first-line options. For refractory or heavily pretreated disease, whole-microbiota FMT may remain relevant because of its broader ecological and metabolic complexity, although its benefit in this setting remains uncertain. For immunosuppressed or high-infection-risk patients, sterile filtrates or inactivated engineered probiotics could represent potentially safer alternatives by reducing the risk of bacteremia, but their clinical efficacy remains to be demonstrated. The foundational question—whether live bacterial colonization is required for therapeutic benefit—will help determine how these product categories should be positioned in future UC treatment strategies. The FRESCO trial and related studies represent early steps toward addressing this question, but their findings will require confirmation before next-generation microbiota therapies can be incorporated into routine clinical practice.

### 5.6 Real-world implementation barriers

Even if precision microbiota strategies prove effective in clinical trials, their translation into routine practice will face important implementation barriers. First, cost and accessibility may limit scalability. Donor–recipient matching based on metagenomics, metabolomics, and machine-learning prediction could be expensive and may remain confined to specialized centers unless simplified, lower-cost tools and regional donor-bank networks are developed. Second, regulatory and manufacturing standardization remain unresolved. FMT and live biotherapeutic products occupy an intermediate space between biologics, fecal-derived materials, and personalized microbial therapeutics, making conventional requirements for product uniformity difficult to apply. Consensus frameworks such as the second ROME consensus (72) provide useful guidance, but cross-national harmonization and enforceable release criteria are still lacking.

Patient acceptability and shared decision-making also require greater attention. The use of donor-derived fecal material, uncertainty regarding donor characteristics, and privacy issues related to donor metagenomic data may influence patient willingness to undergo FMT. Future trials should therefore incorporate implementation science from the outset, including cost-effectiveness modeling, regulatory pathway analysis, standardized product-quality metrics, and patient-reported outcomes. Without addressing these practical barriers alongside efficacy and safety, the transition from empirical FMT to precision microbiota therapy may remain difficult despite scientific progress.

## 6 Limitations of this review

This review has several limitations. First, it is a narrative rather than systematic review, and no formal risk-of-bias assessment or meta-analysis was performed. Second, the mechanistic evidence reviewed here is heterogeneous, and many pathways are supported primarily by animal models, *in vitro* systems, or associative human data. Third, rapidly evolving evidence from recent clinical trials and microbiota-based therapeutics may continue to reshape the field. These limitations reinforce the need for prospective, mechanism-driven, and biomarker-stratified studies of FMT in UC.

Several additional limitations are related to microbiome and multi-omics methodology. Microbiome findings across FMT studies are difficult to compare because sequencing platforms, sequencing depth, reference databases, taxonomic classifiers, strain-resolution pipelines, and bioinformatic workflows continue to evolve. Metabolomic results are also affected by differences in sample processing, analytical platforms, metabolite panels, normalization methods, and timing of sample collection. These methodological differences may partly explain why responder-associated taxa, engraftment signatures, and metabolomic changes are not always reproducible across studies. Publication bias is another concern, as positive FMT trials, responder analyses, and mechanistic associations may be more likely to be reported than negative engraftment or metabolomic findings. Finally, microbiome outcomes are inconsistently defined and reported, with substantial variation in stool versus mucosal sampling, sampling time points, donor-strain engraftment definitions, metabolite readouts, and statistical correction for multiple testing. Future FMT trials should therefore adopt standardized microbiome, metabolomic, and immune-reporting frameworks to improve comparability and strengthen causal interpretation.

## 7 Conclusions and future perspectives

FMT has emerged as a potential microbiota-targeted induction strategy for selected patients with mild-to-moderate UC, particularly when delivered through lower gastrointestinal routes and using optimized or multi-donor preparations. However, current evidence does not support FMT as a uniform or standard therapy for all patients with UC. Its efficacy appears to be shaped by disease severity, delivery route, donor composition, treatment intensity, co-interventions, and recipient ecological receptivity. Recent boundary-defining trials, including RESTORE-UC and STOP-Colitis, underscore that FMT outcomes depend not only on donor quality but also on the reversibility of the recipient inflammatory and ecological environment.

Mechanistically, FMT provides a unique interventional model for understanding microbiota–host ecosystem repair. Available evidence links FMT to microbiota reconstitution, SCFA production, bile acid remodeling, tryptophan–AhR signaling, epithelial barrier regulation, and mucosal immune rebalance. Nevertheless, most mechanistic observations remain associative, and the field still lacks rigorous evidence distinguishing causal mediators from biomarkers of inflammation resolution. Future trials should therefore incorporate longitudinal multi-omics profiling, causal mediation analysis, direct functional readouts of barrier integrity, standardized manufacturing parameters, and long-term safety monitoring.

The next phase of FMT research should move from empirical transplantation toward precision microbiota therapy. This will require prospective validation of donor–recipient matching algorithms, biomarker-guided patient selection, rational sequencing or combination with biologics, and comparative evaluation of whole FMT, defined live biotherapeutic products, and postbiotic or cell-free approaches. Implementation challenges—including cost, donor bank infrastructure, regulatory classification, product standardization, and patient acceptability—should be addressed alongside efficacy and safety rather than after clinical proof-of-concept has been established.

In conclusion, FMT should currently be viewed as an investigational, mechanism-informative, and potentially personalized microbiota-based strategy for selected patients with UC, rather than an established standard therapy. Its greatest contribution may ultimately lie not only in its direct clinical effect, but also in accelerating the development of predictable, durable, and safe precision microbiota therapeutics.
